# Sweet Chestnut (*Castanea sativa* Mill.) Nutritional and Phenolic Composition Interactions with Chestnut Flavor Physiology

**DOI:** 10.3390/foods11244052

**Published:** 2022-12-14

**Authors:** Maria João Santos, Teresa Pinto, Alice Vilela

**Affiliations:** 1 University of Trás-os-Montes and Alto Douro, 5000 801 Vila Real, Portugal; 2 CITAB, Centre for the Research and Technology of Agro-Environmental and Biological Sciences and Inov4Agro, Institute for Innovation, Capacity Building and Sustainability of Agrifood Production, Department of Biology and Environmental, School of Life and Environmental, University of Trás-os-Montes and Alto Douro, 5000 801 Vila Real, Portugal; 3 CQ-VR, Chemistry Research Center, Department of Biology and Environmental, School of Life and Environmental, University of Trás-os-Montes and Alto Douro, 5000 801 Vila Real, Portugal

**Keywords:** sweet chestnuts, chemical composition, flavor, health benefits, qualitative and sensory methods, gastronomy

## Abstract

The European chestnut (*Castanea sativa* Mill.), is an environmentally and economically important species in Europe, mainly for fruit production. The chestnut fruit is well-known for its nutritional properties, namely its high concentration of carbohydrates (starch) and its low-fat content, as well as being one of the few fruits that do not contain gluten. Due to its chemical and nutritional characteristics beneficial to health, the sweet chestnut is a food recommended at different levels. The biochemistry of the mouth and nose of a human being is very complex. However, understanding the different interactions between the biochemistry of our sensory organs and food helps us to comprehend certain concepts, such as flavor and how it is involved in the sensory evaluation of the chestnuts. For the selection of high-quality products, it is necessary to develop reliable methods both from a qualitative and sensory point of view, and chestnut is a fruit with unique sensory characteristics that can be used in various gastronomic dishes, from main courses to desserts.

## 1. Introduction

The chestnut tree belongs to the genus *Castanea* and family *Fagaceae*, the same family as the oaks (*Quercus*) and the beeches (*Fagus*) [1,2,3,4]. This family is widely distributed in the forests of the Northern Hemisphere’s temperate regions. The different species of the genus *Castanea* can be found in China (*Castanea mollissima* BL. and *Castanea seguinii* Dode.), Japan (*Castanea crenata* Sieb. & Zucc.), North America (*Castanea dentata* (Marsh.) Brokh), and Europe (*Castanea sativa* Mill.) [1]. The chestnut tree derives from the presence and influence of humans from prehistoric times to the present day. Thus, there is a long history of culture and tradition around chestnuts, especially those from Southern Europe [5]. In the Middle Ages, chestnut was used as the main ingredient in bread production [6], and in periods of food crisis with a scarcity of food resources, the chestnut was fundamental in the food supply [7,8], especially in the 18th century, which is said to have been the worst famine century in history [9,10]. 

**Figure 1:**
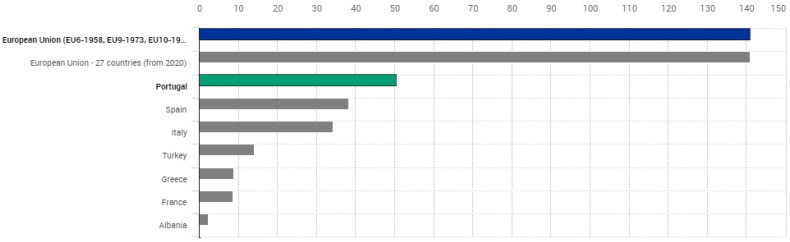
Crop production in EU standard humidity. Chestnut annual area (cultivation/harvested/production) (1000 ha). Source: Eurostat. Adapted from Statistics|Eurostat (europa.eu) [14].

The chestnut tree is a species of great interest in multifunctionality, associated with several agricultural and forestry crops. It presents remarkable biodiversity and a capacity to adapt to different ecosystems [10]. The *Castanea sativa* Mill. is one of the most important fruits in the world and is traditional in the European Mediterranean countries [4,11]. Chestnut production has been increasing worldwide over the last few years, as has the area occupied by chestnut trees, and their productivity. This increased interest in the planting of chestnut trees is largely a result of the increased knowledge of tastemakers about the nutritional qualities of the chestnut and its potential health benefits [4,7]. In addition, there is a growing concern in the chestnut sector, namely in the implementation of the plantation of new chestnut areas. Considering climate change, the new chestnut plantations aim to use plants resistant to diseases that cause high mortality in chestnut groves, such as the disease (*Phytophthora cinnamomi*) and the use of smart irrigation systems installed [12,13]. In 2021, Portugal was the main producer in Europe and represented about 35.7 % of the European production. Indeed, the production in Portugal reached 50.37 thousand tons that year [14]; see Figure 1.

Chestnut (*C. sativa*) is an important resource in Europe due to its economic value associated with fruit, wood, and tannin production, and indirectly with honey production, but also due to its cultural value [4]. Even more, the chestnut tree is known as “tree bread” since its nuts, with elliptical shape, bright reddish-brown color with long dark lines, and intense flavor [7], contain essential nutrients in the human diet, such as starch, sugar, and protein [15].

This review aims to summarize the chemical composition and the nutritional characteristics of chestnuts and their influence on the biochemistry of flavor. Moreover, how to use nuts to produce healthy and tasty foods and/or culinary dishes will also be observed.

## 2. Chestnut Chemical Composition

Chestnuts have played an essential role in human nutrition since ancient times [16]. Nowadays, their integration into the human diet is highly recommended [17]. The main compound of the chestnut is water, where the moisture content ranges from 40 and 64 g/100 g fresh weight [18]. Studies conducted on chestnuts’ chemical and nutritional composition confirm that this fruit is low in fat, cholesterol-free, and gluten-free. On the other hand, it is a rich source of starch (carbohydrates), protein, dietary fiber, vitamins, minerals (such as potassium, phosphorous, and magnesium), lipids, and nutrients. Furthermore, it is a good source of antioxidants such as *L*-ascorbic acid, carotenoids, and phenolic compounds such as gallic and ellagic acids [2,15,16,17,18,19]. Due to the mentioned characteristics, the chestnut may have beneficial health effects, arousing consumer interest. Consumer awareness will require the development of reliable methods to select high-quality foods, both nutritionally and sensorily [19]. The nut’s nutritional value depends on its chemical composition, and this is the result of the interaction of the cultivar (genotype), environmental conditions (climatic factors, temperature, and radiation), and production practices (soil mineral composition, nutrient availability, diseases, and pest) [16,17,20,21].

Nutritionally, chestnuts have interesting characteristics, containing significant amounts of dietary fiber but small amounts of crude protein (2–4%) and low levels of crude fat (predominated by unsaturated fatty acids) (2–5%) compared to typical walnuts (walnuts, almonds, hazelnuts), thus being a good source of energy with multiple health-beneficial effects [4]. On a dry-matter basis, the main components of the chestnut are carbohydrates (75–91%), most notably starch (39–82%), followed by sucrose [18,22]. These polysaccharides, together with glucose, fructose, and raffinose—which are also found in significant quantities—can contribute to the identification of a specific chestnut cultivar [11,16,20,23,24,25]. Researchers argue that starch is partially hydrolyzed into glucose during storage, giving chestnuts a higher sweetness [18]. Moreover, chestnuts with a higher starch content are more suitable for flour production [16]. The content and composition of these sugars are influenced by various conditions such as storage temperature, relative humidity, harvest time, oxygen level, and even the packaging itself [20]. Chestnuts with lower moisture content may have a longer shelf life after harvest because they are likely to have a less favorable environment for the development of microorganisms [26]. As previously referred, the chestnuts are also low in fat, thus helping to decrease cholesterol levels. Additionally, they are rich in vitamin C, macro- (K, P, Mg, Ca, Na), and micronutrients (Mn, Fe, Zn, and Cu) [4,27]. The mineral content found in chestnut trees is associated not only with the genotype and the climatic conditions but also with the mineral composition of the soil where the chestnut trees were grown [21]. Chestnuts also have a significant antioxidant activity associated with polyphenolic and organic contents [4]. The composition of chestnuts, in different processing, estimated by the United States Department of Agriculture [28], confirmed that chestnuts are rich in starch, minerals, vitamins, and phytonutrients, and low in calories and fat (Table 1). Borges [26] studied the chemical composition of eight sweet chestnut cultivars (Aveleira, Reborda, Trigueira, Zeive, Demanda, Longal, Martaınha, and Judia) from three protected designation of origin (PDO) areas located in the Trás-os-Montes region of Portugal. The results once again proved that chestnuts contain high levels of starch (43 g/100 g dry matter) and low fat (3 g/100 g dry matter). In addition, they are a good source of minerals, containing potassium (≈750 mg/100 g dry matter), phosphorus (≈120 mg/100 g dry matter), and magnesium (≈75 mg/100 g dry matter); and amino acids (6–9 g/100 g dry matter) [26]. Additionally, there are differences depending on the type of processing the chestnut is subjected to, Table 1. The way chestnuts are processed—roasting or cooking—has effects on their primary and secondary metabolic composition. In roasted chestnuts, there is a higher protein, insoluble, and total dietary fiber content [21]. When the chestnut is cooked, significant changes occur in the macromolecular structure of the starch (important polysaccharide), modifying its digestibility and making it more bioavailable. In addition, cooked chestnuts are a good source of phenolic compounds and organic acids and are low in fat, properties that are associated with positive health benefits [21,29]. 

## 3. Chestnut Nutritional and Health Benefits

Nowadays, the consumer has been showing more attention to the characteristics of the products he consumes, prioritizing healthy food. The chestnut presents a wealthy chemical and nutritional composition capable of altering the food quality. Studies conducted on Portuguese cultivars have revealed that chestnuts contain small amounts of crude fat, are low in saturated fatty acids, and high in unsaturated fatty acids. Chestnut fat contains monounsaturated fatty acids (MUFA), which are known for their anticancer effects and their association with a reduced risk of sudden death that is related to cardiovascular diseases and disorders of neurological function [21]. In addition to the fat properties, the chestnut also contains natural phenols and flavonoids with antioxidant activity to prevent cardiovascular disease. These components are essential in preventing diabetes and several types of cancers [4,15]. Regarding digestive health, nuts reduce cholesterol levels and stabilize blood sugar levels. Furthermore, the phospholipids in the chestnuts are associated with high antioxidant activity, and linoleic acids play an essential role in preventing cardiovascular disease in adults and promoting brain and retinal development in children [30]. The energy value (gross energy) in nuts, as in any other food source, comes from the oxidative breakdown of carbohydrates, proteins, and lipids. Not all the gross energy released is available to the body, however, the available energy is vital for metabolic mechanisms allowing the maintenance and thermoregulation of the body [21]. The chestnut is a nutritional supplement in the human diet and contains carbohydrates necessary for short-term or long-term energy [21,31]. Therefore, starch is the main bioavailable carbohydrate in the human diet and provides the energy needed for the catabolism process of amylose and amylopectin into glucose. In addition, it has a positive role in intestinal functions [18,25]. According to Choupina [32], the chestnut is rich in carbohydrates (approximately 40 g/100 g of edible product) and has a starch content of approximately 27 g/100 g of edible product. An amylose/amylopectin ratio of European chestnut starch has also been observed, such that it could have some nutritional importance due to its beneficial effects in preventing diabetes and allergies. Vitamins are essential for a balanced and healthy diet. Chestnuts contain fat-soluble vitamins (Ascorbic acid, B1, B2, B3, B6, and E) which promote healthy skin and improve brain function [15,33]. They play a key role in the aging process, help prevent blood clotting, are gluten-free, and are of great benefit to patients with celiac disease [4,34,35]. In addition, they also contain vitamin C (ascorbic acid) [15,32], which is an important antioxidant for human colon cells [36]. The intake of minerals is equally crucial for the proper functioning of the human body. Calcium (Ca) is associated with essential biological functions such as ensuring the proper functioning and rigidity of the skeleton. Magnesium (Mg) is an important cofactor for many enzymes and is involved in protein synthesis and maintaining cell membranes’ electrical potential. Microminerals such as iron are essential for oxygen transport and in several enzymes that participate in the synthesis and catabolism of many nutrients [21,37]. Cooking nuts can form potentially toxic compounds, such as acrylamide formation during high-temperature cooking. In addition to developing a darker color, roasted chestnuts have considerable amounts of acrylamide that can have adverse health effects, being classified as a probable human carcinogen and also known as neurotoxins [38]. Behind the proprietaries of the chestnuts as fruit, their processing chain generates a huge number of residues such as shoots, shells, and leaves. These residues—generally discarded—are rich in bioactive compounds. Chestnut leaves, for example, are used in infusions in folk medicine to cure coughs and diarrhea [31,39]. Chestnuts can be used as functional ingredients and natural preservatives. Over time, medicinal products based on herbs (powders, extracts, essential oil) and nuts have been obtained to protect the health of the population [3].

## 4. Chestnut Sensory Pleasantness

The chestnuts are rarely consumed raw, being processed in several ways, at home or industrially, to improve their organoleptic characteristics (aroma, flavor, and texture). The processing of the chestnut for consumption, as well as improving the flavor characteristics, improves the digestibility of the fruits, i.e., makes the nutrients more bioavailable and increases the shelf life of several industrial process products [21].

### 4.1. Chestnut Taste and Flavor

From an early age, taste and aroma have been known to go together in one sense [40,41]. The combination of both leads to the recognition of the most varied sensory experiences to which human taste buds are constantly subjected. Thus, along with the sensations felt by the trigeminal nerve, the concept of “flavor” is created. The flavor is defined by the International Organization for Standardization [42] as “a complex sensation that originates from the combination of taste, olfactory, and trigeminal sensations perceived during the tasting of a product”. It can also be seen as a different “sense” that is cognitively constructed from other senses—taste, touch, and smell—to identify foods and beverages [43,44,45,46]. 

Despite the great diversity of chemical substances that are associated with food, the taste is nothing more than a set of five basic sensations: salty, acid, sweet, umami, and bitter [47,48,49,50]. In addition to these sensations, a sixth sensation has recently been identified, called fat taste [49].

**Figure 2:**
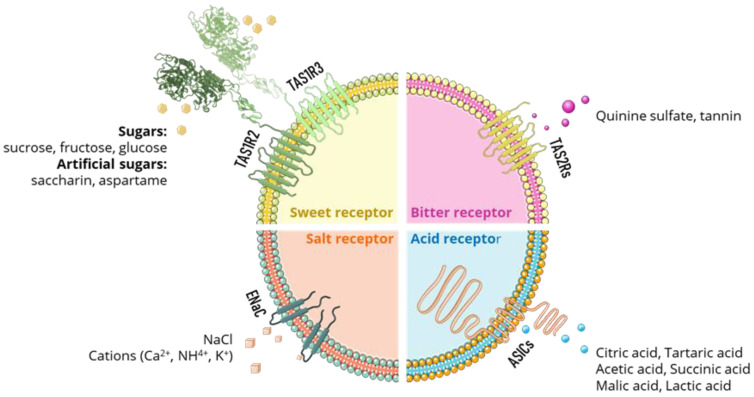
Examples of natural stimuli and taste receptors are involved in the recognition of four taste modalities—sweet, salty, sour, and bitter.

The human taste system is composed of sensory organs called taste buds, which are located in the tongue. The taste buds have the function of analyzing the chemical compounds of the food/beverage that comes into contact with the receptor cells during its consumption [50]. Sugars and sweeteners are sensed by the TAS1R2/ TAS1R3 receptor family. Salt and different cations are sensed with the help of ion channels, one of which is the ENaC receptor. Bitter compounds such as quinine sulfate and tannin are sensed by a set of 25 taste receptors, called TAS2Rs. The detection of acidic molecules such as citric acid, lactic acid, and others, is accomplished through receptors such as ASICs (Figure 2)

**Figure 3:**
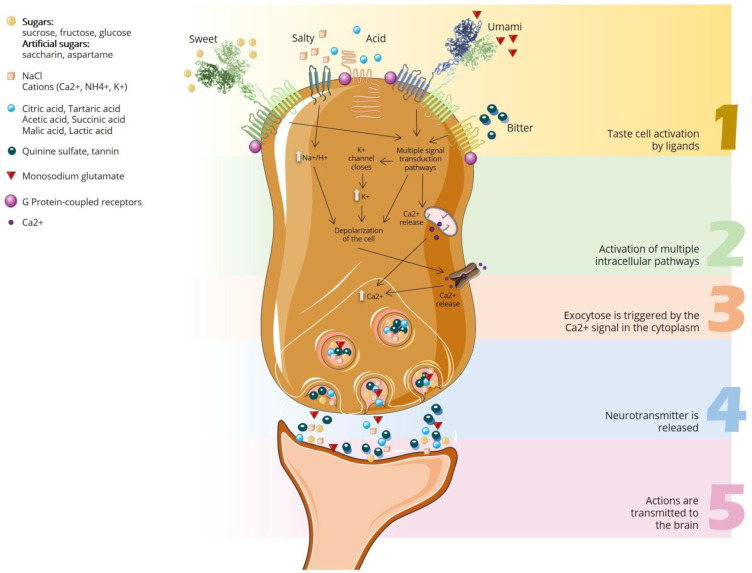
Generalized scheme of taste transduction. Tastants bind to specific receptors on the apical membrane. All stimuli elicit increases in intracellular Ca^2+^, either by activation of voltage-gated Ca^2+^ channels or by causing the release of Ca^2+^ from intracellular stores.

The signal transduction mechanism of taste begins with the presence of chemical compounds (tastants) which produce changes in the taste receptor cells of the taste buds by force of the various chemoreceptors, namely the G protein-coupled receptors [51] (Figure 2) that constitute them. These receptors are found primarily on the apical microvilli of the taste cells and the transduction machinery involves ion channels on both the apical and basolateral membranes, creating action potentials in the nerve fibers. Channels typically found in axonal membranes are located on the basolateral aspect of taste cells. These include voltage-gated Na^+^, K^+^, and Ca^2+^ channels that produce depolarizing potentials when taste cells interact with chemical stimuli. The resulting receptor potentials raise Ca^2+^ to levels sufficient for synaptic vesicle fusion and synaptic transmission, thus eliciting action potentials in the afferent axons (Figure 3). In general, the greater the tastant concentration, the greater the depolarization of the taste cell [52].

The human diet’s most abundant source of the salty taste is sodium chloride (NaCl) [47,53,54]. The latter, together with potassium chloride (KCl), give rise to the sodium and potassium ions (Na^+^; K^+^), determinants for the maintenance of various physiological processes. Based on the diuretic effect of amiloride, several studies [53,54,55,56] have shown that there are at least two mechanisms responsible for the detection of salty taste: the amiloride-sensitive pathway (AS) and the amiloride-insensitive pathway (AI). These two mechanisms differ in their sensitivity to certain salts and the fact that they are mediated by different cells in the taste bud. The AS pathway is a highly Na^+^ selective pathway and is responsible for mediating appetite behavioral responses when confronted with low to moderate NaCl concentrations. The salt receptor used is the epithelial sodium channel (ENaC), which is a channel that is strongly sensitive to the inhibitory action of amiloride and confers high specificity to the Na^+^ ion. The activation of taste cells by the AS pathway occurs when there is an increase in the concentration of Na^+^ in saliva and these ions enter the taste cells directly via the ENaC channels. This is followed by depolarization of the cell membrane that will generate an action potential. As a consequence, depolarization causes the opening of the calcium homeostasis modulator (CALHM1/3) channels and the release of neurotransmitters and ATP (adenosine triphosphate) in the afferent nerve fibers [53,54,55,56]. The mechanism of detection by the amiloride insensitive (AI) pathway is not yet well known, however, it is believed that humans have another group of “insensitive” taste cells capable of mediating salty taste at high concentrations [55]. Salt is a common additive in starch-based products, such as chestnuts. Salt improves the organoleptic properties and modifies the rheological properties of the product where it is used [57,58]. The most common salt additives are sodium chloride and calcium chloride [58]. Taste depends not only on the concentration added but also on the type of salt used [57].

Considered the “nutritional guardian” of the human body, acid taste helps prevent the consumption of certain foods/beverages that are unfit for consumption and may be harmful to humans, such as green, spoiled, or fermented foods. The presynaptic/type III cells detect acid taste modality. Over time, several genes have been proposed to function as receptors in acid taste detection, including the ASICs gene (acid-sensitive ion channels) [48,59,60] and, more recently, OTOP1 (Otopetrin 1) [60,61]. The stimulus of acid taste is caused by the increased concentration of H+ ions on the surface of the tongue and promotes an ionic flow into the taste cells through the taste receptors, as mentioned above. This flow will be responsible for the opening of calcium channels (Ca^2+)^ and, consequently, the depolarization of the cell membrane and the release of neurotransmitters in the afferent nerve fibers [53]. Finally, the impulse is transmitted to the taste center of the central nervous system to initiate the perception of the sour taste. Some authors have stated that raw chestnuts have a higher malic acid content than cooked chestnuts [29]. Malic acid, although a weak acid, contributes to the flavor of the nut, and the presence or absence of it in different processing methods can cause differences in consumer taste. As with most vitamins, ascorbic acid has a sour taste profile and generally does not serve to improve the flavor of food. On the other hand, some say that fruit jams and jellies benefit from this acidity, by it giving a touch of freshness to the products. Sweet and umami tastes are often confused or thought of as the same taste. This confusion stems from the fact that these two tastes, despite having different characteristics, share the same transduction system and have a common receptor. For a sweet taste, sucrose is the reference substance [62]. However, there are other sweet-tasting compounds, such as sugars (glucose, fructose, raffinose, etc.), artificial sweeteners (saccharin, aspartame, and others), sweet amino acids, and proteins, and some plant metabolites [62,63].

As for the umami taste, which means “delicious,” the main substance linked to this sensation is L-glutamate—an amino acid found in the form of monosodium glutamate (MSG) [43,44,47,50,64]. Sweetness detection in the oral cavity of the taster occurs in type II cells and is mediated by a heterodimer receptor called T1R2/T1R3 that is coupled to the G protein superfamily [44,62,63,65,66]. The transduction mechanism of the umami taste modality is very similar to the sweet-taste mechanism [67,68,69]. The prominent taste receptor is also a heterodimer belonging to the G-protein coupled receptors but formed by T1R1/T1R3 proteins [44,64,68,70,71]. Several studies have revealed that medium/high concentrations of monosodium glutamate increase the perception of salty taste, modulate that of sweet taste, and suppress the perception of sour and bitter tastes [71,72]. The high percentage of starch and sucrose are among the most important parameters for evaluating the quality of the chestnut and are directly related to the sweet taste and texture [18,21,23]. These characteristics are decisive for the consumers’ appreciation of the different cultivars of chestnuts [23]. The sugars present in the chestnut have an essential role in this fruit’s color, taste, and odor. They participate in the Maillard chemical reaction, which occurs during the chestnut roasting, and which is responsible for most of the sensorial characteristics of the roasted chestnut [5].

The bitter taste is imparted through a wide variety of chemical structures, such as salts, alkaloids (such as quinine), flavonoids, amino acids, polyphenols, and others [47,62,73]. As with the elemental sweet and umami tastes, the transduction mechanism of the bitter taste is mediated by receptors that belong to the family of taste receptors coupled to the G protein and that are identified in type II receptor cells. In humans, and in particular, for bitter tastes, this family of receptors is composed of about 25 receptors that are called “Taste Receptor Family 2”—T2Rs [68,73,74,75,76,77,78]. As with the elemental sweet and umami tastes, the interaction between the receptor and, subsequently, the G-protein is critical for an increase in PLCβ2 activity and, consequently, the IP3R3 receptor to be activated; an event that mediates the release of intracellular Ca^2+^ [63,65,66]. The last mechanism in the transduction concerns the activation of the TrpM5 ion channel, a consequence of the calcium increase. The opening of this channel will cause membrane depolarization and the release of ATP to adjacent cells that will be responsible for communicating and stimulating afferent nerve fibers [63,65,66]. 

In the interesting review work performed by Hu et al. [79], about the bioactive phenolic components and potential health effects of the chestnut shell, it is possible to perceive the complex constitution of chestnuts concerning all the phenolic compounds present namely in chestnut shells. Some of those compounds, namely phenolic acids, flavonoids, and tannins, give the chestnut its perceived bitterness and astringency. Furthermore, rutin and quercetin are flavonoids with inhibitory activity against α-amylase [80], which may cause some inhibitory effects on the sweet-taste perception ability. With the inhibition of the salivary α-amylase activity, it is not possible to cleave the starch molecule, allowing glucose to be free and sensory perceptive.

Recently Pinto and co-workers [81] found that the tannin content in *C. sativa* was higher than that of phenolic acids and flavonoids. However, the quality and quantity of phenolic compounds in *C. sativa* are certainly dependent on the variety and geographical origin. Vella et al. [82] measured the ellagic acid in the outer shells and inner skins of four varieties of chestnuts in Italy and found that the Tempestiva variety had the highest amount of ellagic acid. The results showed that the content of ellagic acid in the outer shells was about 0.9 mg/g dry weight, and the inner skins presented an amount of 1.38 mg/g dry weight. Silva et al. [83] observed total phenols in the inner and outer shells, burs, and leaves of the Portuguese *Castanea sativa* Mill. Were, respectively, 321 ± 3; 240 ± 6; 242.4 ± 0.9 and 385.4 ± 0.5 µg of epicatechin equivalents/mg of residue, and the content of tannins, for the same variety by-products, were respectively, 35 ± 5; 9 ± 1; 5.5 ± 0.4 and 113 ± 1 µg of epicatechin equivalents/mg of residue.

So, we can infer that cooking chestnuts, with the inner and outer shells, by boiling them or roasting them, will have a significant impact on the chestnuts’ overall taste and flavor. Moreover, the culinary process will also impart health benefits once the phenolic compounds present in the inner shell seem to be effective against multidrug-resistant bacteria and can even be used as coadjutors to antibiotics [79,83].

Another compound family that may transmit some bitterness of the chestnut is amino acids because they are substances present in the chestnut and responsible for some chemical transformations that occur during the different processes [21].

Although it was only recently identified as the sixth elemental taste, the fat taste was already considered different from the other elemental basics. The stimuli responsible for the taste of fat are the breakdown products of fats and fatty acids [84]. Studies indicate that the CD36 transporter, protein, which is found in taste buds, specifically in the circumvallate and leaf papillae [85], and the G protein-coupled receptor (GPCR) is the most likely candidate receptors in taste cells, with various taste transduction mechanisms also involved [86]. Chestnut fruit includes relatively low unsaturated fatty acids (oleic, linoleic, and palmitic acids) [8]. Studies indicate that roasting chestnuts decreases the concentration of unsaturated and saturated fatty acids. Moreover, lipids strongly influence the final aroma of the chestnut [5,87]. Cooked chestnuts are low in fat, and these properties are also associated with positive health benefits [21].

### 4.2. Trigeminal Sensations and Chestnut Palatability

Trigeminal sensations or “mouthfeel” are defined as “a group of sensations that is characterized by the tactile response in the mouth” [88] and are also described as “tactile properties (sensation) perceived from the time food or drink-solid, semi-solid, or liquid-is placed in the mouth until it is swallowed” [89]. The filiform papillae are responsible for the perception of trigeminal sensations [48,88]. These papillae have no taste receptors and play an important role in responding to mechanical, thermal, and nociceptive stimuli [48,88]. These stimuli interact with the trigeminal nerve and give rise to the oral cavity’s physical, chemical, and textural sensations [82]. The trigeminal sensory nerve fibers have a variety of chemosensory receptors that encode various sensations in the mouth, such as astringency, burning, viscosity, temperature, body, tingling, and metallic sensation, among others [88,90]. 

Although the nuts are consumed after being peeled, they are processed together with the shell. As can be seen in Table 2, the volatile compounds are affected by the peeling of the varieties. Just as it affects the aroma, the shell can influence the flavor of the seed. The chestnut shell is a rich source of biologically active compounds, especially phenolic compounds [91]. According to Hu et al. [79], the tannin content in chestnut shells is higher than that of phenolic acids and flavonoids, and condensed tannins are the main components of chestnut shell extract.

Tannins are then one of the most important bioactive components present in sweet chestnuts and affect the consumer’s palatability due to their bitter taste and astringency. The roasting process, in addition to improving the color and flavor of the nut, reduces the effect of tannins and improves its digestibility and preservation [2]. However, other chestnut tree tissues, such as leaves, wood, and bark, have much higher levels of these phenolics [21,93].

### 4.3. Olfactory Sensations and Chestnut Aroma

The olfactory system is responsible for activating many chemical stimuli, giving us information about their origin, concentration, and quality.

Odor molecules from foods/beverages are detected through olfactory receptors, which are responsible for the transduction of volatile odors and located in the olfactory epithelium [94,95]. Olfactory receptors can detect multiple odorant molecules, and each can bind to multiple chemoreceptors, meaning that the perception and identification of odors coming from the combination of the various receptors activated when stimulation occurs [41,96]. Thus, the olfactory system can detect and discriminate a large number of scents. The olfactory epithelium detects odor molecules in the nasal cavity via one of two pathways: (1) the orthonasal pathway, which involves the molecules being picked up directly by the nose [41,48,94], and (2) the retronasal pathway, which concerns the molecules detected after entering the oral cavity and contacting the back of the throat [41,48]. Chestnuts have a relatively short shelf life, due to the high-water activity and sugar content. Chestnuts are preferably consumed roasted or cooked, either fresh and freshly harvested or frozen and, later, consumed out of season [29]. Cooking chestnuts most often leads to positive changes in aroma and flavor. Typically, the aroma of chestnut is more associated with products made from chestnut and not the fresh fruit itself. However, there are studies on the volatile compounds present in fresh peeled and unpeeled chestnuts (Table 2).

The study of Mujić et al. [92] on the volatile composition of the raw chestnut aroma resulted in the identification of quite a few volatile compounds. The data in Table 2 showed that the chemical compositions of the volatile compounds were strongly affected by the peeling of the chestnuts. 

On the other hand, during this fruit’s roasting and cooking processes, Maillard reactions occur between amino acids and reducing sugars, which leads to the formation of volatile heterocyclic compounds involved in flavor [5,21]. This process may also be responsible for modifying polysaccharides and proteins in the nut after cooking. Volatile compounds previously identified in the study of Kris and co-workers [97] in roasted chestnuts including γ-butyrolactone (mild, sweet, caramel flavor) and furfural (sweet, woody, almond, fragrant, cooked flavor) [21]. 

Food is an essential resource for human survival. For this reason, the ability to identify a flavor is a factor of enormous relevance in the choice and consumption of different food products. The sweet chestnut is no exception; the flavor of this nut is a consequence of its chemical composition and interactions with the consumer’s oral cavity.

## 5. Methods and Sensory Lexicons Used on Chestnut Sensory and Qualitative Evaluation

The tasting panel is the most widely used tool when it comes to sensory analysis and has been the preferred tool for many studies to draw reliable conclusions about the sensory characteristics of chestnuts. Quantitative descriptive analysis (QDA) method is the most famous for the sensory analysis of chestnuts or chestnut-based products. QDA is a technique that has been widely used to quantify and optimize sensory attributes where the sensory panel is trained to identify and quantify sensory attributes using appropriate intensity scales so that statistical analysis can be performed [98].

This method has been used for more than two decades. Beginning in 1999 until some years later, Künsch and his team used QDA analysis to test the quality of the product made of chestnuts (the purée) and of roasted chestnuts [5,99]. Both studies used a panel of more than 35 tasters and an intensity scale from 1 to 10. The attributes analyzed were, among others, taste, sweetness, aroma, and texture. In 2010, Castro-Vázquez [100], and Carneiro-Carvalho [101], 2019, also used the QDA method. The first [100] evaluated the quality of chestnut honey using a 0 to 10 to rate the intensity of each attribute. In this case, the scales were delimited by the terms “weak” and “strong”. In the second study [101] randomly harvested chestnuts after different treatments were applied to them while they were still on the mother tree. To quantify the sensory attributes, the tasters used a structured scale from 1 (less intense) to 5 (most intense) points, according to the ISO 4121 reference [102].

The analysis of food for consumption is becoming more and more rigorous. As a consequence, and with the advancement of technology, there are non-destructive methods capable of evaluating the characteristics of foods and thus which are important allies to sensory analysis. Recent publications have proven the potential of near-infrared spectroscopy (NIR) as a rapid and non-destructive method for agro-food analysis [27,103,104]. Moreover, this technique has been widely used for the quality assessment of nuts. For example, a study conducted in 2021 [19] with sensory and qualitative analysis of chestnuts, used data fusion strategies, taking advantage of the synergistic effect of the information obtained from sensory analysis of chestnuts with the help of the panel and QDA test and the data obtained by NIR. This study showed that sweet chestnuts, due to their characteristics, have the potential to be used in secondary products such as jam and flour, as mentioned before. In other studies protein content and sugar content in chestnuts have been measured using NIR spectroscopy [105,106]. In both cases, the authors stated that this method is faster compared to conventional methods and is a non-destructive method [27]. 

## 6. Chestnut in Gastronomy

**Figure 4:**
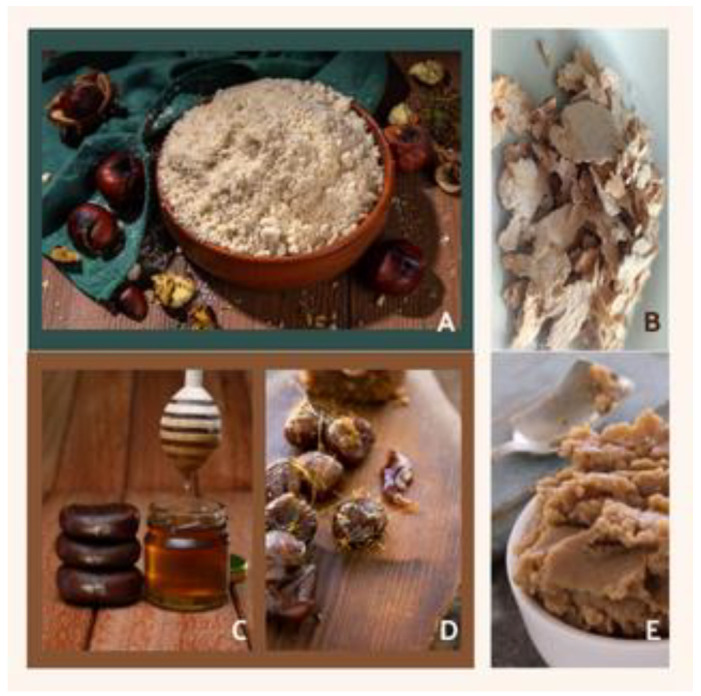
Examples of products made from chestnuts and the chestnut tree. (**A**)—chestnut flour; (**B**)—chestnut flakes; (**C**)—chestnut honey; (**D**)—candied chestnut; (**E**)—chestnut purée.

The composition of nutrients in foods in general and in processed novel foods in particular changes for various reasons. These reasons may be biological origins, such as plant variety, pre-harvest cultural practices, and seasonal and annual factors. On the other hand, they may be related to differences in post-harvest, storage, and processing [107]. In recent years, consumers have preferred healthy, fresh, and high-quality food, and chestnuts have high amounts of healthy bioactive metabolites and nutritional qualities, capable of improving the performance of the human body [3,8,26]. Moreover, chestnuts are very versatile when it comes to cooking, they can be present in numerous dishes or consumed alone as a snack. Sweet chestnut varieties are generally classified according to their geographical origin and morphological and phenological characteristics. They can be divided into several groups depending on the primary use of their fruits; some varieties produce chestnuts that are ideal for fresh and sweet commercialization, while others are more suitable for drying and flour production [8,22,31,39]. Chestnuts can be consumed in their fresh state, raw, boiled, or roasted, or as a processed ingredient, such as chestnut flour, chestnut flakes, purée, yogurts, or candied chestnuts; see Figure 4 [8,31,39,108]. 

Roasting of the chestnuts is well accepted by tasters because of their sweetness, coming from the sucrose content. The chestnuts for roasting should be selected according to their size, peelability, sucrose content, and typical aroma [27]. In the production of traditional and candied chestnuts, as a seasonal product, the fruits are selected, and the small-sized ones are removed from the processing line for not being suitable for production [15]. The “marrons glacées” are highly appreciated in France, Italy, Switzerland, and Spain. In the production of these sweet browns, the fruits are infiltrated or submerged in a sugar-rich solution and then covered with sugar syrups, and then the fruits are baked in a 300 °C oven for 1–2 min for the sugar to crystallize. The chestnut has to be in optimal condition after storage and has a good caliber [3,8,21,109]. Chestnut honey is seen as one of the most delicious and high-quality honey, with Spain being one of the main producers of this type of product. The geographical origin of the chestnut trees is a relevant factor that can affect the volatile composition of the honey and, consequently, the flavor characteristics [100], Figure 1. The combination and transformation of the chestnut with other ingredients is undoubtedly a profitable strategy as it reduces the waste levels in industrial processing and provides a wider range of new chestnut products available to the consumer [8,15,107]. There are many examples of processing and combining nuts with other products. The well-known chestnut flour has grown as an ingredient in gluten-free diets, being incorporated into pasta production [3,34]. Even more, some chestnuts are put in increasing sugar concentrations and placed in a jar, and others are added to alcoholic beverages—such as brandies, cognacs, or liqueurs—to sweeten them for a period of 6 to 12 months [21]. Another study evaluated the nutritional change of freeze-dried roasted Chinese chestnuts after chocolate coating and tested their quality during storage [107]. The results were positive, and the chocolate-coated chestnut was considered a new product. The chocolate coating led to a marked reduction in the population of spoilage microorganisms during storage and showed satisfactory bacteriological quality. Another non-seasonal product idea with an extended shelf life is the production of chestnut pickles [15]. Pickling is one of the oldest methods of food preservation. It is gaining much more importance and guarantees food safety and shelf-life extension [110]. In this situation, the chestnuts are the basis for the production of pickles, and salt, acetic acid, and garlic are used as pickling agents [15].

## 7. Final Remarks

Over the past years, there has been a growing demand for traditional foods with functional and nutritional value. The chestnut is a unique fruit—even compared to wheat and rice—and Portugal has the privilege of being one of the largest producers of this nut. The taste and sensations transmitted by the chestnut are singular and vary according to their previously undergone process. Thus, there are several products made from chestnuts with unique characteristics. For the analysis of these products, we used sensorial and chemical analysis with the help of different techniques.

## 8. Future Perspectives

Future research directions could focus on sensory analyses and sensory profiles of the different chestnut cultivars, given the few studies on this subject. Moreover, the biochemical interaction between the chestnut and the taster’s oral cavity is not explored in detail. In the future, an understandable relation between the chestnuts’ chemical properties and our mouth’s chemical proprieties could be an added value for choosing a product made from this nut and the refinement request by the consumer.

## Figures and Tables

**Table 1:** Nutritional value of 100 g of European chestnut; Source: National Nutrient Database for Standard Reference, United States Department of Agriculture; Retrieved from FoodData Central (usda.gov) [28].

	Nutrient Value		
	Raw	Roasted	Boiled
Energy (Kcal)	196	245	131
General composition (g)			
Water	52	40.5	68.2
Protein	1.63	3.17	2
Total lipid (fat)	1.25	2.2	1.38
Fatty acids, total saturated	0.235	0.414	0.26
Fatty acids, total monounsaturated	0.43	0.759	0.476
Fatty acids, total polyunsaturated	0.493	0.869	0.545
Carbohydrates	44.2	53	27.8
Fiber, total dietary		5.1	
Sugars, total including NLEA ^1^		10.6	
Vitamins			
Folates (µg)	58	70	38
Niacin (mg)	1.1	1.34	0.731
Pantothenic acid (mg)	0.476	0.554	0.316
Riboflavin (mg)	0.016	0.175	0.104
Thiamin (mg)	0.144	0.243	0.148
Vitamin A (IU)	26	24	17
Vitamin C (mg)	40.2	26	26.7
Electrolytes (mg)			
Sodium	2	2	27
Potassium	484	592	715
Minerals (mg)			
Calcium	19	29	46
Cooper	0.418	0.507	0.472
Iron, Fe	0.94	0.91	1.73
Magnesium	30	33	54
Manganese	0.336	1.18	0.854
Phosphorus	38	107	99
Zinc	0.49	0.57	0.25

^1^ Total sugar on the Nutrition Labelling and Education Act (NLEA).

**Table 2:** The concentration of volatile compounds calculated by peak area (%) by solid phase microextraction (SPME) headspace analysis of triplicate extractions for shelled and unshelled raw chestnut seeds. Adapted from Mujić et al. [92].

	Compound Name	Flavor Description	Concentration Calculated by Peak Area (%)	
			Peeled Chestnuts	Unpeeled Chestnuts
Alcohols				
	Ethanol	Alcoholic	20.61 ± 1.02	14.19 ± 2.57
	2-Propanol	Butter	ND	1.64 ± 0.57
	1-Propanol	Alcohol, apple, musty, fruity, peanut, pear	ND	0.34 ± 0.08
	2-Methyl-1-propanol	Fruity, Wine-like	ND	3.57 ± 0.31
	2-Pentanol	Oily, green	ND	0.76 ± 0.19
	3-Methyl-1-butanol	Oily, whiskey	ND	5.27 ± 0.83
	2-Methyl-1-butanol	Fuel oil, sweet	0.75 ± 0.13	2.86 ± 0.59
	1-Pentanol	Sweet, vanilla	1.54 ± 0.26	0.55 ± 0.09
	2,3-Butanediol	Neutral sensory characteristics	2.46 ± 0.11	ND
	1-Hexanol	Green, herbaceous, woody	6.90 ± 1.38	2.82 ± 0.56
	2-Heptanol	Oily, earthy	0.76 ± 0.19	0.69 ± 0.16
	1-Octen-3-ol	Cheese, creamy, earthy, herbaceous	ND	2.32 ± 0.45
	3-Octanol	Melon, musty, oily	0.54 ± 0.13	5.02 ± 0.23
	1-Octanol	Citrus, fatty, woody	18.40 ± 4.05	13.06 ± 1.72
	Phenylethyl alcohol	Honey, rose	0.74 ± 0.19	0.91 ± 0.41
	Total alcohols		52.80 ± 4.66	54.00 ± 2.31
Aldehydes				
	Octanal	Honey, fruity, fatty, citrus	0.61 ± 0.22	ND
	Benzaldehyde	Almond, cherry, sweet	0.58 ± 0.19	0.41 ± 0.10
	Total aldehydes		1.19 ± 0.36	0.41 ± 0.10
Ketones				
	Acetone	Apple, ethereal	2.97 ± 1.01	1.45 ± 0.27
	2-Pentanone	Alcohol, apples, banana, cheese	0.87 ± 0.19	1.35 ± 0.27
	2,3-Pentanedione	Buttery, cheesy, sweet, nutty, fruity	0.72 ± 0.09	0.62 ± 0.17
	3-Hydroxy-2-butanone	Sweet, buttery, creamy, dairy	ND	0.87 ± 0.09
	2-Heptanone	Banana, cinnamon, spicy, fruity	5.86 ± 1.14	2.37 ± 0.49
	2-Octanone	Green, herbaceous, floral, fruity	0.91 ± 0.18	ND
	6-Methyl-5-hepten-2-one	Oily, herbaceous, green	0.60 ± 0.09	ND
	Total ketones		11.93 ± 2.03	6.66 ± 0.44
Esters				
	Methyl acetate	Ethereal, sweet	1.27 ± 0.29	0.45 ± 0.08
	Ethyl 2-methyl propanoate	Sharp, sweet, green, apple, fruity	ND	0.64 ± 0.14
	Ethyl butyrate	Tropical fruit, tutti-fruity, mango flavor	0.84 ± 0.07	0.45 ± 0.05
	3-Methylbutyl acetate	Fruity, pear, banana-like odor	0.75 ± 0.10	ND
	2-Methylbutyl acetate	Banana, peanut	0.72 ± 0.06	0.72 ± 0.16
	Methyl octanoate	Fruity, green, citrus	ND	2.95 ± 0.39
	Octyl acetate	Jasmine, herbaceous, fruity	1.01 ± 0.22	0.38 ± 0.06
	Total esters		4.59 ± 0.35	5.59 ± 0.77
Terpenoids				
	Limonene	Lemon, orange, citrus, sweet	1.73 ± 0.21	0.84 ± 0.46
	Total terpenoids		1.73 ± 0.48	0.84 ± 0.24
Acids				
	Acetic acid	Strong, pungent sour odor	11.01 ± 2.10	4.28 ± 0.85
	Hexanoic acid	Cheese, fatty, sour	1.39 ± 0.34	ND
	Total acids		12.40 ± 2.57	4.28 ± 0.93
Furans				
	Tetrahydrofuran	Ether-like	0.57 ± 0.07	ND
	2-Pentylfuran	Green bean, metallic, vegetable	10.26 ± 0.26	2.02 ± 0.55
	Total furans		10.83 ± 1.39	2.02 ± 0.41
